# Effect of endodontic access design and coronal restoration on fracture resistance

**DOI:** 10.6026/973206300223202

**Published:** 2026-05-31

**Authors:** Amireddi Venkata Siva Durga Prasad, Gembali Sandeep Kumar, Purna Sai Prasad Kolavali, Srikrishna Muppalaneni, Sai Tejaswi G, Chavali Kavya Yadav

**Affiliations:** 1 Department of Conservative Dentistry and Endodontics, KLR Lenora Institute of Dental Sciences, Rajahmundry, India; 2 Department of Conservative Dentistry and Endodontics, Sri Balaji Dental College, Moinabad, India; 3 Department of Pediatric and Preventive Dentistry, SVS Institute of Dental Sciences, Mahabubnagar, India; 4 Department of Dentistry, Fortune Dental, Vijayawada, India; 5 Department of Conservative Dentistry & Endodontics, Fortune Dental, Vijayawada, India; 6 Department of Oral Medicine & Radiology, Prime Dental Care, Gudivada, India

**Keywords:** Endodontics, fracture resistance, access cavity design, dental restoration, primary teeth, permanent teeth, biomechanics

## Abstract

Endodontically treated teeth are prone to fracture due to structural loss following access cavity preparation and restoration. Therefore, it is of interest to evaluate the effect of access cavity design and coronal restoration on fracture resistance and prosthetic suitability in 120 extracted primary molars and permanent premolars. Conservative access designs showed significantly higher fracture resistance than traditional designs. The crown restorations outperformed composite restorations in both dentitions (p < 0.05). Teeth restored following conservative access showed greater prosthetic suitability, with higher ferrule preservation compared to traditional access designs. Thus Conservative access cavities combined with full-coverage restorations optimize biomechanical performance and prosthetic prognosis in both primary and permanent teeth.

## Background:

Endodontic treatment remains a cornerstone intervention for preserving teeth affected by pulpal pathology. However, the structural consequences of access cavity preparation, root canal instrumentation and subsequent loss of dental hard tissues significantly compromise the biomechanical integrity of treated teeth [1, 2]. Epidemiological studies indicate that endodontically treated teeth demonstrate a 2.8-fold increased risk of fracture compared to vital counterparts, with fracture representing the primary cause of post-treatment tooth loss [3]. The traditional endodontic access cavity philosophy emphasized unobstructed visualization and straight-line access to root canal systems, often necessitating extensive removal of coronal tooth structure, including critical load-bearing dentin in the peri-cervical region [4]. Contemporary minimally invasive endodontic approaches advocate for conservative access cavity designs that preserve maximal peri-cervical dentin while maintaining adequate canal negotiation and cleaning efficacy [5, 6]. Coronal restoration strategy constitutes another critical determinant of long-term success for endodontically treated teeth. The selection between direct composite restorations, indirect restorations and full-coverage crowns depends on multiple factors including remaining tooth structure, occlusal loading and esthetic requirements [7, 8]. While full-coverage crowns have traditionally been recommended for posterior endodontically treated teeth, recent evidence suggests that conservative restorations may be appropriate when adequate tooth structure remains [9, 10]. The differential biomechanical properties of primary versus permanent teeth introduce additional complexity in treatment planning. Primary teeth exhibit lower mineral content, thinner enamel and more pronounced pulp chambers relative to crown dimensions, potentially influencing their response to access cavity preparation and restoration [11]. Therefore, it is of interest to evaluate the effect of endodontic access design and coronal restoration on fracture resistance.

## Materials and Methods:

## Specimen selection and preparation:

One hundred twenty extracted human teeth were collected from oral surgery departments and private dental practices. Inclusion criteria specified: (1) primary mandibular second molars or permanent mandibular second premolars, (2) intact crowns without caries, cracks, or previous restorations, (3) fully developed roots with closed apices for permanent teeth and (4) extraction within the previous three months. Teeth were stored in 0.1% thymol solution at 4°C until experimental procedures commenced. Teeth underwent cleaning with ultrasonic scalers to remove soft tissue remnants and calculus. Digital radiographs were obtained in mesiodistal and buccolingual projections to confirm structural integrity and exclude internal resorption or calcifications. Root dimensions were standardized within ±1.5 mm using a digital calliper. Specimens were then randomly allocated into six experimental groups for each dentition type (n=10 per group) using computer-generated randomization sequences.

## Endodontic access cavity preparation:

Access cavities were prepared under 4.5x magnification (dental loupes) using round carbide burs (#2, #4) and tapered diamond burs operating at high speed with copious water irrigation. Three distinct access designs were implemented:

## Traditional access cavity (TAC):

Outline to achieve straight-line access to all canal orifices with complete deroofing of the pulp chamber. For primary molars, access extended from mesial marginal ridge to distal marginal ridge. For premolars, access encompassed the central groove with extension toward buccal and lingual cusps as needed.

## Conservative access cavity (CAC):

Minimally invasive preparation preserving peri-cervical dentin and roof structures while maintaining canal identification and instrumentation feasibility. Access opening reduced by 3204

approximately 40% compared to traditional design, with preservation of chamber roof dentin between orifices.

## Truss access cavity (TrAC):

Modification of traditional access maintaining a dent in bridge (truss) between mesial and distal canal orifices, creating separate mesial and distal access openings.

## Root canal treatment:

Following access preparation, working lengths were established using electronic apex locators and confirmed radiographically at 0.5 mm short of the radiographic apex. Canal preparation utilized nickel-titanium rotary instrumentation (ProTaper Gold system, Dentsply Sirona) to size F3 for primary teeth and F4 for permanent teeth, with copious 2.5% sodium hypochlorite irrigation between instruments. Final irrigation protocols included: 5 mL 17% EDTA (2 minutes), 5 mL 2.5% sodium hypochlorite and 5 mL sterile saline. Canals were dried with paper points and obturated using continuous wave vertical compaction technique with gutta-percha and AH Plus sealer (Dentsply Sirona). Access cavities were temporarily sealed with Cavit-G (3M ESPE) for 48 hours to allow sealer setting.

## Coronal restoration procedures:

Following temporary filling removal, specimens received either direct composite restorations or ceramic crown preparations:

## Direct composite restoration:

Access cavities were etched with 37% phosphoric acid gel (20 seconds dentin, 30 seconds enamel), rinsed and gently air-dried. A universal adhesive system (Single Bond Universal, 3M ESPE) was applied according to manufacturer instructions. Nanohybrid composite resin (Filtek Z350 XT, 3M ESPE) was placed incrementally in 2 mm layers with light polymerization (1200 mW/cm^2^, 20 seconds per layer). Final restorations reproduced anatomical contours and were finished with fine diamond burs and polishing discs.

## Ceramic crown restoration:

Teeth were prepared for full-coverage ceramic crowns with 1.5 mm occlusal reduction, 1.0 mm axial reduction and 1.0 mm circumferential chamfer finish line positioned 1.0 mm coronal to the cementoenamel junction. Preparations maintained 4 mm occlusal convergence angle and preserved at least 2 mm ferrule height. Polyvinyl siloxane impressions were obtained and lithium disilicate crowns (IPS e.max CAD, Ivoclar Vivadent) were fabricated using CAD/CAM technology. Crowns were cemented with dual-cure resin cement (RelyX Ultimate, 3M ESPE) following manufacturer protocols including hydrofluoric acid etching and silane application. Control group specimens remained intact without endodontic treatment or restoration. All restored specimens were stored in distilled water at 37°C for 7 days before mechanical testing to simulate oral hydration conditions.

## Specimen mounting and thermocycling:

Root surfaces were coated with polyether impression material (0.3 mm thickness) to simulate periodontal ligament compliance. Specimens were then embedded in auto-polymerizing acrylic resin using custom cylindrical molds (25 mm diameter x 30 mm height), with the cementoenamel junction positioned 2 mm coronal to the resin surface to simulate physiological bone levels. Mounted specimens underwent thermocycling (5,000 cycles between 5°C and 55°C, 30-second dwell time, 10-second transfer time) to simulate aging processes and thermal stress accumulation.

## Fracture resistance testing:

Fracture resistance was evaluated using a universal testing machine (Instron 5960, Instron Corporation) equipped with a 10 kN load cell. A stainless steel spherical indenter (6 mm diameter) was positioned to contact the occlusal surface at the central fossa, oriented perpendicular to the long axis of the tooth. Compressive loads were applied at a crosshead speed of 1 mm/min until catastrophic failure, defined as an abrupt drop in load-displacement curve or audible crack indicating fracture. Maximum fracture load (Newtons) was recorded for each specimen. Load-displacement curves were analyzed to determine stiffness values (N/mm) calculated from the linear portion of the curve prior to fracture.

## Fracture pattern analysis:

Following mechanical testing, fractured specimens were visually examined under stereomicroscope (20x magnification) to classify fracture patterns according to established criteria:

[1] Type I (Favorable): Fracture confined to crown structure above the cementoenamel junction

[2] Type II (Questionable): Fracture extending to the cementoenamel junction without subgingival propagation

[3] Type III (Unfavorable): Fracture propagating more than 2 mm apical to the cementoenamel junction

[4] Type IV (Catastrophic): Vertical root fracture or fracture extending beyond middle third of root

## Prosthetic suitability assessment:

Post-fracture evaluation included measurement of remaining supragingival tooth structure using digital calipers. Prosthetic suitability was determined based on established criteria: (1) presence of at least 1.5 mm ferrule height, (2) tooth structure circumferentially surrounding preparation margin and (3) absence of catastrophic fractures precluding restoration. Specimens meeting all criteria were classified as "suitable for prosthetic rehabilitation".

## Statistical analysis:

Statistical analyses were performed using SPSS Statistics version 27.0 (IBM Corporation). Normality of data distribution was confirmed using Shapiro-Wilk tests. Descriptive statistics including means and standard deviations were calculated for fracture loads, stiffness values and ferrule measurements. One-way analysis of variance (ANOVA) assessed differences in fracture resistance among experimental groups within each dentition type, followed by post-hoc Tukey honest significant difference tests for pairwise comparisons. Two-way ANOVA evaluated interactions between access cavity design and restoration strategy. Chi-square tests compared fracture pattern distributions and prosthetic suitability proportions among groups. Statistical significance was established at α=0.05 for all analyses.

## Results and Discussion:

**Figure 1:**
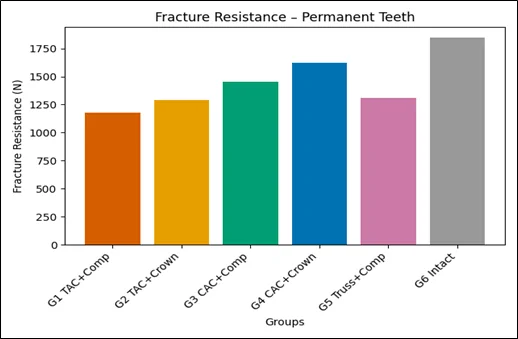
Mean fracture resistance (N) of permanent teeth across different access cavity designs and restorative strategies.

**Figure 2:**
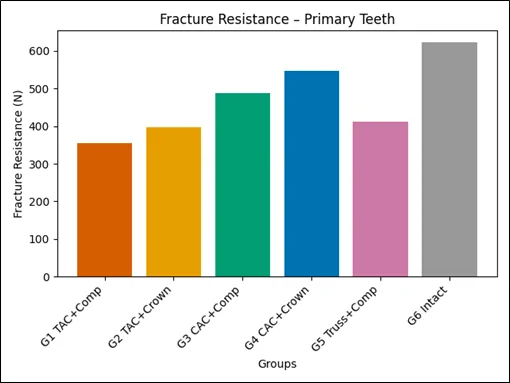
Mean fracture resistance (N) of primary teeth across different access cavity designs and restorative strategies.

Fracture resistance varied significantly according to access cavity design and coronal restoration strategy in both dentitions. In permanent premolars, intact teeth provided the structural benchmark, demonstrating the highest resistance (1847 ± 156 N). Among treated groups, conservative access followed by crown restoration yielded the best performance (1623 ± 142 N), whereas traditional access combined with direct composite showed the lowest resistance (1178 ± 119 N), corresponding to a reduction of approximately 36% compared with sound teeth (F = 156.3, p < 0.001). The distribution of these values is presented in Figure 1, Table 1. The highest fracture resistance was observed in the conservative access with crown group, while traditional access with composite showed the lowest values (Figure 1). Primary molars demonstrated the same hierarchy despite lower absolute loads (F = 134.2, p < 0.001). Conservative access restored with crowns maintained nearly 88% of intact strength (548 ± 76 N versus 623 ± 87 N), while traditional access with composite retained only 57% (Figure 2). The reproducibility of this ranking across dentitions indicates that fracture behavior is governed primarily by how much strategically important dentin remains rather than by tooth type itself. A similar trend was observed in primary teeth, with conservative access preparations demonstrating greater resistance (Figure 2). Across restorative approaches, conservative cavities consistently outperformed traditional preparations. Within composite groups, conservative access improved fracture resistance by 23.6% in permanent teeth and 36.8% in primary teeth (p < 0.001). Comparable advantages were observed in crown-restored specimens. These outcomes emphasize the mechanical value of peri-cervical dentin, a region widely described as a structural buttress critical for stress dissipation and resistance to crack propagation. Loss of this area increases cuspal flexure, shifts tensile stresses apically and predisposes to vertical or non-restorable fractures [12]. Crown placement provided additional reinforcement in all access designs, increasing fracture resistance by approximately 9-13%. This confirms the stabilizing influence of cuspal coverage and ferrule creation. However, crowned traditional preparations still did not achieve the performance of conservative cavities, indicating that prosthetic intervention cannot completely reverse Truss designs demonstrated intermediate resistance (permanent: 1312 ± 125 N; primary: 412 ± 64 N). The preserved dentin bridge likely improved stress distribution compared with traditional access, yet incomplete peri-cervical conservation prevented equivalence with conservative preparations. The overall pattern therefore suggests a continuum in which greater tissue preservation results in progressively better biomechanical outcomes [14]. Two-way ANOVA verified significant effects for both access design and restoration type, together with interaction between them, in permanent and primary teeth. Stiffness measurements paralleled fracture resistance values, with conservative preparations demonstrating reduced displacement before failure (p < 0.001), further supporting enhanced rigidity. Fracture pattern analysis clarified the clinical importance of these numerical differences. Strong associations existed between treatment strategy and failure mode (permanent: ^2^ = 78.3; primary: ^2^ = 72.1; p < 0.001). Conservative access preparations predominantly resulted in fractures confined to the crown or limited to the CEJ, leaving most teeth potentially restorable. In contrast, traditional access frequently produced subgingival or vertical root fractures. Within traditional composite groups, catastrophic failure occurred in 40% of permanent teeth and 50% of primary teeth. Crown placement favorably altered this distribution, reducing the frequency of severe root involvement. Nevertheless, the most predictable pattern remained conservative access combined with cuspal coverage, where both resistance and reparability were highest. Truss cavities again displayed intermediate behaviour (Table 2). Post-fracture prosthetic evaluation revealed perhaps the most clinically meaningful observation. Conservative access preserved adequate ferrule and circumferential tooth structure in the large majority of specimens. In permanent teeth, 87% remained suitable for crown rehabilitation compared with only 54% of traditional preparations; primary teeth showed nearly identical differences. Residual ferrule height was almost double in conservative groups, greatly enhancing the feasibility of future restorative intervention [15, 16, 17, 18]. Specimens originally restored with crowns also demonstrated slightly better rehabilitation potential after failure, likely reflecting more favourable redistribution of stresses during loading. However, preservation of dentin at the time of access preparation appeared to be the dominant determinant of whether retreatment remained possible [19, 20]. The convergence of fracture load, fracture pattern and ferrule preservation indicates that structural conservation governs not only resistance to failure but also the biological cost of failure when it occurs. While laboratory conditions cannot reproduce cyclic fatigue and oral aging, the uniformity of these trends across analyses suggests that the advantage of conservative access is fundamental [21, 22, 23].

## One-way ANOVA:

Permanent teeth: F=156.3, p<0.001; Primary teeth: F=134.2, p<0.001

## Prosthetic suitability:

Conservative vs Traditional, ^2^=14.8, p<0.001 (permanent); ^2^=13.2, p<0.001 (primary)

N = Newtons; SD = Standard Deviation; n=10 per group

## Chi-square analysis:

Permanent teeth: ^2^=78.3, p<0.001; Primary teeth: ^2^=72.1, p<0.001

## Conclusion:

Conservative endodontic access cavity designs significantly improve fracture resistance and prosthetic suitability in both primary and permanent teeth by preserving peri-cervical dentin. Crown restorations provide additional fracture resistance, particularly in traditionally accessed teeth, though conservative access maintains superior long-term restorative options. These findings support conservative access combined with appropriate coronal restoration as an evidence-based approach to optimize structural integrity and future prosthetic flexibility.

## Study limitations and future directions:

Several limitations warrant consideration in interpreting these findings. The *in vitro* design, while enabling controlled variable manipulation, does not replicate the complex biological environment including pulpal healing responses, dentinal fluid dynamics and bacterial presence that may influence clinical outcomes. The single-load static fracture testing methodology does not simulate cyclic masticatory fatigue, which represents the predominant clinical loading pattern and may preferentially compromise certain restorative approaches. Our specimen selection criteria excluded teeth with existing restorations or structural anomalies, potentially limiting generalizability to more complex clinical scenarios. The standardized root canal preparation to specific sizes and the use of continuous wave obturation technique, while ensuring consistency, may not represent all clinical endodontic protocols. The seven-day water storage period and 5,000 thermocycle aging protocol provide limited simulation of long-term oral environmental exposure. Future investigations should incorporate cyclic loading protocols simulating masticatory fatigue over extended timeframes. Clinical longitudinal studies comparing conservative versus traditional access approaches with adequate follow-up periods (5-10 years) would provide definitive evidence regarding real-world performance. Advanced imaging modalities including micro-computed tomography could quantify three-dimensional structural changes and relate them to biomechanical outcomes. Finite element modelling validated against experimental data could enable predictive analyses for individual clinical scenarios. The interaction between access cavity design and specific restoration materials (various composite formulations, ceramic types, fibre-reinforced composites) merits systematic investigation. The influence of tooth position (molar versus premolar, maxillary versus mandibular), root canal anatomy complexity and operator experience on outcomes requires elucidation. Investigation of minimally invasive access approaches in teeth with calcified canals or complex anatomy would address important clinical scenarios.

## Advancement to knowledge:

This study demonstrates that preservation of peri-cervical dentin through conservative access designs significantly enhances fracture resistance and post-fracture restorability. It further establishes that coronal restoration strategy cannot fully compensate for structural loss caused by aggressive access preparation. The combined evaluation of fracture resistance, fracture pattern and prosthetic suitability provides clinically relevant insight into long-term treatment planning.

## Funding statement:

Nil

## Figures and Tables

**Table 1:** Mean fracture resistance and prosthetic suitability across experimental groups

**Group**	**Access Design**	**Restoration**	**Permanent Teeth**	**Primary Teeth**	**Prosthetic Suitability**
			**Mean ± SD (N)**	**Mean ± SD (N)**	**Permanent/ Primary**
Group 1	Traditional	Composite	1178 ± 119	356 ± 52	50% / 40%
Group 2	Traditional	Crown	1289 ± 135	398 ± 58	60% / 60%
Group 3	Conservative	Composite	1456 ± 128	487 ± 69	80% / 80%
Group 4	Conservative	Crown	1623 ± 142	548 ± 76	90% / 90%
Group 5	Truss	Composite	1312 ± 125	412 ± 64	70% / 60%
Group 6	Control (Intact)	None	1847 ± 156	623 ± 87	-

**Table 2:** Fracture Pattern Distribution by Treatment Group: Type I: Favorable (crown only); Type II: Questionable (at CEJ); Type III: Unfavorable (>2mm subgingival); Type IV: Catastrophic (vertical root fracture)

**Group**	**Access Design**	**Restoration**	**Type I**	**Type II**	**Type III**	**Type IV**	**Favorable/Questionable**
**Permanent Teeth**							
Group 1	Traditional	Composite	2 (20%)	1 (10%)	3 (30%)	4 (40%)	30%
Group 2	Traditional	Crown	3 (30%)	2 (20%)	3 (30%)	2 (20%)	50%
Group 3	Conservative	Composite	5 (50%)	3 (30%)	2 (20%)	0 (0%)	80%
Group 4	Conservative	Crown	6 (60%)	2 (20%)	2 (20%)	0 (0%)	80%
Group 5	Truss	Composite	3 (30%)	3 (30%)	3 (30%)	1 (10%)	60%
**Primary Teeth**							
Group 1	Traditional	Composite	1 (10%)	2 (20%)	2 (20%)	5 (50%)	30%
Group 2	Traditional	Crown	2 (20%)	3 (30%)	3 (30%)	2 (20%)	50%
Group 3	Conservative	Composite	4 (40%)	3 (30%)	2 (20%)	1 (10%)	70%
Group 4	Conservative	Crown	5 (50%)	3 (30%)	1 (10%)	1 (10%)	80%
Group 5	Truss	Composite	3 (30%)	2 (20%)	3 (30%)	2 (20%)	50%
